# Improving of the Photovoltaic Characteristics of Dye-Sensitized Solar Cells Using a Photoelectrode with Electrospun Porous TiO_2_ Nanofibers

**DOI:** 10.3390/nano9010095

**Published:** 2019-01-12

**Authors:** Min Su Jo, Jung Sang Cho, Xuan Liang Wang, En Mei Jin, Sang Mun Jeong, Dong-Won Kang

**Affiliations:** 1 Department of Engineering Chemistry, Chungbuk National University, 1 Chungdae-ro, Seowon-Gu, Cheongju, Chungbuk 28644, Korea; jms039@naver.com; 2 Department of Chemical Engineering, Chungbuk National University, 1 Chungdae-ro, Seowon-Gu, Cheongju, Chungbuk 28644, Korea; wangxuanleon@gmail.com; 3 School of Energy Systems Engineering, Chung-Ang University, Seoul 06974, Korea; kangdwn@cau.ac.kr

**Keywords:** electrospinning, nanocomposites, porous TiO_2_ nanofiber, light harvesting, additive, dye-sensitized solar cells

## Abstract

Porous TiO_2_ nanofibers (PTFs) and dense TiO_2_ nanofibers (DTFs) were prepared using simple electrospinning for application in dye-sensitized solar cells (DSSCs). TiO_2_ nanoparticles (TNPs) were prepared using a hydrothermal reaction. The as-prepared PTFs and DTFs (with a fiber diameter of around 200 nm) were mixed with TNPs such as TNP-PTF and TNP-DTF nanocomposites used in photoelectrode materials or were coated as light scattering layers on the photoelectrodes to improve the charge transfer ability and light harvesting effect of the DSSCs. The as-prepared TNPs showed a pure anatase phase, while the PTFs and DTFs showed both the anatase and rutile phases. The TNP-PTF composite (TNP:PTF = 9:1 wt.%) exhibited an enhanced short circuit photocurrent density (*J_sc_*) of 14.95 ± 1.03 mA cm^−2^ and a photoelectric conversion efficiency (PCE, *η*) of 5.4 ± 0.17% because of the improved charge transport and accessibility for the electrolyte ions. In addition, the TNP/PTF photoelectrode showed excellent light absorption in the visible region because of the mountainous nature of light induced by the PTF light scattering layer. The TNP/PTF photoelectrode showed the highest *J_sc_* (16.96 ± 0.79 mA cm^−2^), *η* (5.9 ± 0.13%), and open circuit voltage (*V_oc_*, 0.66 ± 0.02 V).

## 1. Introduction

Dye-sensitized solar cells (DSSCs) are basically thin-layer solar cells consisting of two sandwich-type transparent conduction oxide (TCO) electrodes. One electrode is a highly colored photoelectrode with a few micron-thick layers of mesoporous TiO_2_ or other semiconductors (ZnO, SnO_2_, and Nb_2_O_5_) coated with a photosensitizer, while the other is a Pt-based counter-electrode [1,2,3,4,5]. The space between the two electrodes is filled with an organic electrolyte containing a redox mediator (I^−^/I_3_^−^), usually a mixture of iodine and iodide in organic solvents such as acetonitrile [6,7,8,9,10]. DSSCs, which are cost-effective and have high theoretical efficiencies, are available in various colors depending on the dye. DSSCs co-photosensitized with an organic dye of alkoxysilyl-anchor dye (ADEKA-1, molecular structures of carbazole dyes with a trimethoxysilyl group) and a carboxy-anchor organic dye of LEG4 anchored to nanocrystalline TiO_2_ films show a photoelectric conversion efficiency (PCE) of up to 14.7% (under 1 sun illumination) [11]. The theoretical PCE limit of DSSCs using a simple junction configuration (under standard test conditions) is reported to be 33% [12,13]. However, the PCE of present DSSCs is significantly lower than their theoretical efficiency. The PCE response of DSSCs depends on their open circuit voltage (*V_oc_*, V), photocurrent density (*J_sc_*, mA cm^−2^), and fill factor (*FF*, %) [14,15]. The *V_oc_* is estimated from the difference between the quasi-Fermi levels of semiconductor materials, such as TiO_2_, and the redox potential of the electrolyte (I^−^/I_3_^−^). Therefore, the *V_oc_* of DSSCs is closely related to the properties of the semiconductor materials constituting them. The *J_sc_* and *FF* of DSSCs depend on the adsorption of the dye and the charge transfer in the photoelectrode [15,16,17].

TiO_2_ typically used for a photoelectrode is chemically stable, non-toxic, and readily available in vast quantities [18,19,20,21,22]. For the enhanced photovoltaic properties of TiO_2_ for DSSC, it is imperative to improve its surface area, dye molecular adsorption activity, light harvesting effect, and charge transport. There have been many reports on the use of metal oxide additives, such as SiO_2_, SnO_2_, and ZrO_2_, for improving the photovoltaic properties of TiO_2_ nanoparticles [23,24,25]. In our previous study [26], SnO_2_ hollow fibers were used as the additives for the TiO_2_ photoelectrodes in order to improve the electron transport of DSSCs. The addition of SnO_2_ hollow fibers accelerated the electron transfer and improved the electrochemical properties of the DSSC. The PCE of the DSSC with the SnO_2_ hollow fiber-added TiO_2_ photoelectrode was approximately 11% higher (5.43%) than that of the DSSC with pure TiO_2_ photoelectrode (4.89%). Swathy et al. [27] used nanostructured (≈400 nm particle size) anatase titania spheres as the light scattering layer for DSSCs. These DSSCs showed about two times higher *J_sc_* (10.44 mA cm^−2^) and two times higher PCE (4.92%) than those of TiO_2_ photoelectrodes without scattering layers (*J_sc_* of 5.83 mA cm^−2^, PCE of 2.7%). The photovoltaic performance of the DSSCs was measured under an AM1.5G solar spectrum with a light intensity of 100 mW cm^−2^.

**Scheme 1:**
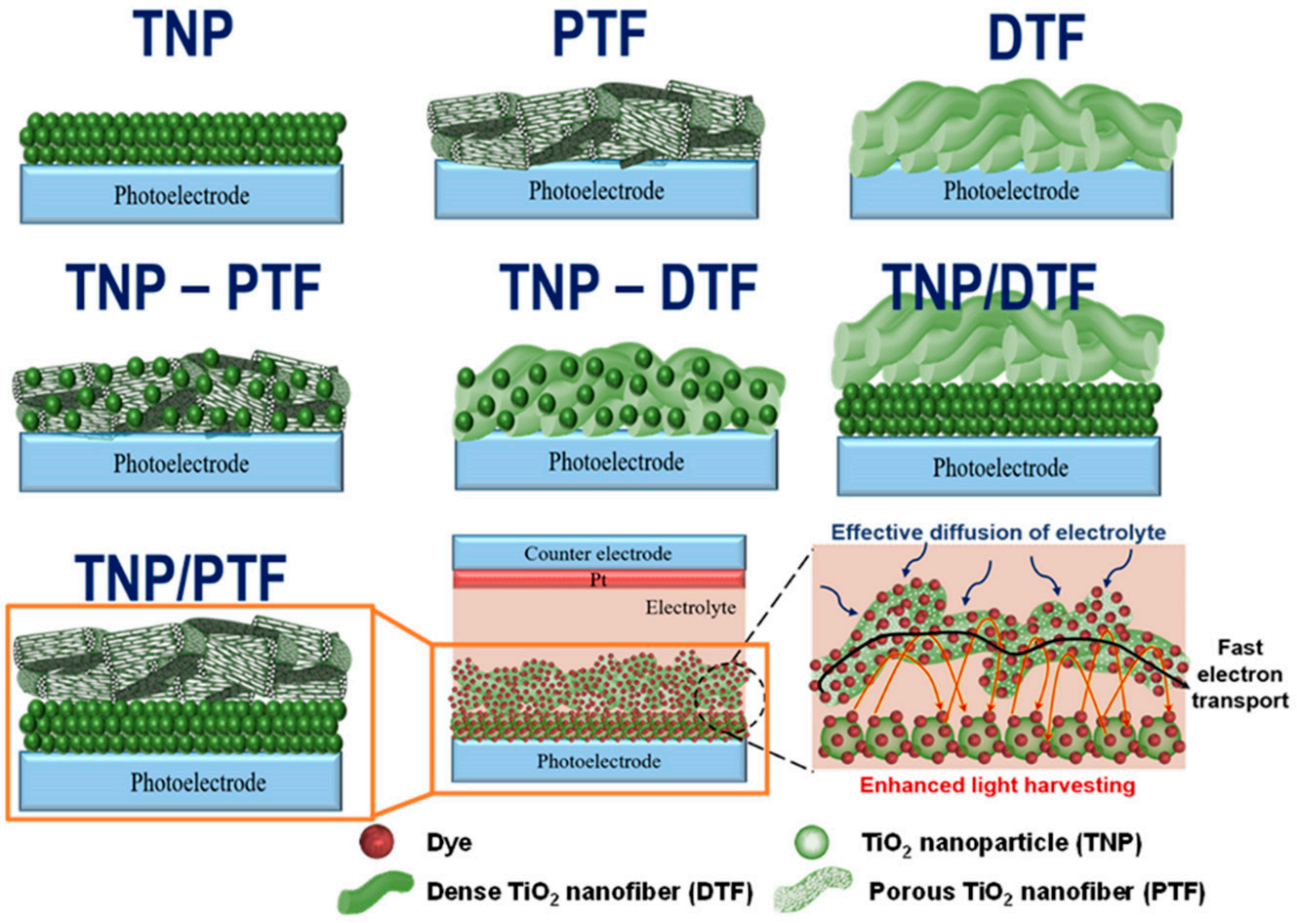
Various photoelectrodes for DSSCs.

In summary, the PCE of DSSCs can be easily improved by altering the design of their photoelectrodes. In this study, we prepared seven TiO_2_ photoelectrodes to improve the PCE of DSSCs, as shown in Scheme 1. The TiO_2_ photoelectrodes were fabricated using TiO_2_ nanoparticles and the porous TiO_2_ nanofibers (PTFs), and dense TiO_2_ nanofibers (DTFs) were used as additives or light scattering layer. The PTFs and DTFs were prepared using simple electrospinning. TiO_2_ nanoparticles (TNPs) were prepared using a hydrothermal reaction and were used for fabricating the photoelectrodes. The as-prepared PTFs and DTFs were used as additives or were coated as light scattering layers to improve the charge transfer ability and light harvesting effect of the DSSCs.

## 2. Materials and Methods

### 2.1. Preparation of Porous TiO_2_ Nanofibers (PTFs)

The porous TiO_2_ nanofibers (PTF) were prepared via an electrospinning process and subsequent heat treatment. The spinning solution was prepared by adding 2 mL butyl titanate (TBT, C_16_H_36_O_4_Ti, ≥97%, Kanto Chemical Co. Inc, Tokyo, Japan), 1 g polyacrylonitrile (PAN, Mw: 150,000, Sigma-Aldrich, St. Louis, MO, USA), 4 g polystyrene (PS, Mw: ≈192,000, Sigma-Aldrich), and 2 mL of acetic acid (99.7%, Daejung Chemicals and Metals, Siheung, Korea) in 40 mL of *N*,*N*-dimethylformamide (DMF, ≥99.5%, Samchun Chemical, Seoul, Korea). The resulting solution was stirred at room temperature for 24 h to ensure complete dissolution and was then loaded into a plastic syringe equipped with a 25 G stainless steel needle (*Ø_in_* = 0.25 mm, *Ø_out_* = 0.51 mm). The needle was connected to a high-voltage supply and a voltage of 20 kV was applied between the needle and the collector. The distance between the needle tip and the drum collector was set at 15 cm at a flow rate of 2 mL h^−1^. The rotation of the drum was maintained at 150 rpm. The resulting electrospun fibers were heated at 100 °C in air for 6 h to remove the solvent, and then heat-treated at 500 °C for 3 h (heating rate was 5 °C min^−1^) in air to remove the polymer.

### 2.2. Preparation of Dense TiO_2_ Nanofibers (DTFs)

The dense TiO_2_ nanofibers (DTF) were also prepared via an electrospinning process and subsequent heat treatment. The spinning solution was prepared by adding 4 mL TBT, 2 g polyvinylpyrrolidone (PVP, M.W. 1,300,000, Alfa Aesar, Haverhill, MA, USA), and 8 mL of acetic acid in 20 mL of ethanol (99.7%, Daejung Chemicals and Metals, Siheung, Korea). The resulting solution was electrospun and heat-treated under the same condition as described above for PTF.

### 2.3. Preparation of Anatage TiO_2_ Nanoparticles (TNPs)

TNPs were synthesized via the hydrothermal method. In a typical reaction, 15 mL of titanium tetraisopropoxide (TTIP, 99.9%, Aldrich) was dissolved in 50 mL of distilled water and the resulting solution was vigorously stirred for a few minutes before adding 0.7 mL of ammonia solution (28–30%, SAMCHUN) to it. This solution was magnetically stirred at 350 rpm for 3 h to obtain a homogeneous mixture. The reaction mixture so obtained was transferred to a Teflon-lined autoclave and was then heated at 200 °C for 5 h. Finally, the white precipitate was washed with distilled water and ethanol and then dried at 80 °C for 12 h.

### 2.4. Preparation of DSSCs

TiO_2_ photoelectrodes were fabricated using TNP or TNP-DTF or TNP-PTF nanocomposites. The TiO_2_ pastes were prepared by mixing acetylacetone (99%, Sigma-Aldrich), hydroxypropyl cellulose (99%, Aldrich), and distilled water. Each paste was coated on a fluorine-doped tin oxide (8 Ω/sq, Pilkington) substrate, which was subsequently sintered at 450 °C for 30 min. The obtained TiO_2_ films were immersed in a 0.5 mM ethanol solution of N719 dye (bis-(isothiocyanato)bis(2,2-bipyridyl-4,4-dicarboxylato)ruthenium(II)bis-tetrabutylammonium, Solaronix) for 5 h. The active area of the photoelectrode was 0.25 cm^2^. The sandwich-type DSSC was assembled using a Pt counter-electrode and a dye-sensitized photoelectrode. A redox I^−^/I_3_^−^ electrolyte was introduced between the electrodes. The preparation methods of the counter electrode and I^−^/I_3_^−^ electrolyte were described in detail in our previous report [17].

In this study, we prepared seven kinds of photoelectrodes, as shown in Scheme 1. The photoelectrodes with only TNP, PTFs, and DTFs were labeled as TNP, PTF, and DTF, respectively. The TNP photoelectrodes with 10 wt.% of PTFs and DTFs were denoted as TNP-PTF and TNP-DTF, respectively. The photoelectrodes with PTF and DTF light scattering layers were denoted as TNP/PTF and TNP/DTF, respectively.

### 2.5. Characteristic and Measurements

The crystalline phases and morphologies of the prepared materials were identified using X-ray diffraction (XRD, Ultima IV, Rigaku, Japan), field-emission scanning electron microscopy (FE-SEM; LEO-1530, Carl Zeiss, Oberkochen, Germany), and field-emission transmission electron microscopy (FE-TEM, 200KV, JEM 2100F, JEOL, Tokyo, Japan). To estimate the specific surface area, pore volume, and pore size distribution of the materials, and their N_2_ adsorption/desorption isotherms were measured at 77 K using a surface area analyzer (ASAP2020, Micromeritics, Norcross, GA, USA). The Brunauer–Emmett–Teller (BET) method and Barrett–Joyner–Halenda (BJH) model were used to estimate the specific surface area and pore size distribution of the samples, respectively. Ultraviolet-visible (UV-Vis-NIR) reflectance spectra were obtained using a UV-Vis-NIR spectrophotometer (Cary 5G, Varian, Palo Alto, CA, USA) equipped with diffuse reflectance accessory (integral sphere). Total reflectance spectrum (Total reflectance = Diffuse reflectance + Specular reflectance) was recorded in the spectral range of 200 to 800 nm at a scan rate of 600 nm min^−1^. In addition, the UV-Vis spectrum of N719 solution was measured using S-3100 equipment (Sinco, Seoul, Korea). The photovoltaic properties of the DSSCs were evaluated by recording their current density–voltage characteristics under illumination from a Polaronix K201 (McScience, Suwon, Korea) equipped with a K401 CW150 lamp power supply and an AM 1.5G filter (100 mW cm^−2^).

## 3. Results

**Figure 1:**
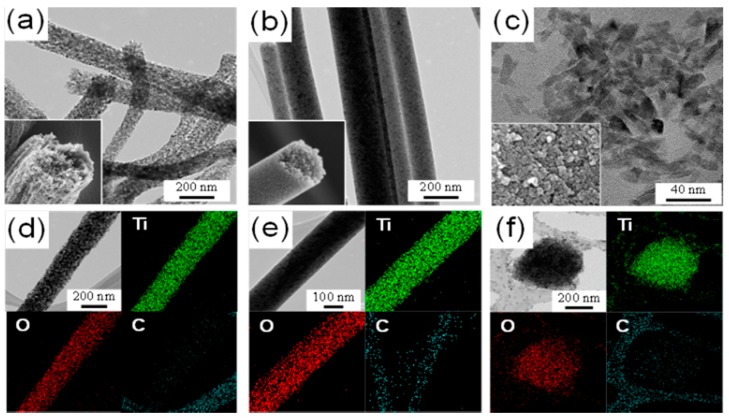
FESEM and FETEM images of the (**a**) PTF, (**b**) DTF, and (**c**) TNP powders. FETEM and EDX mapping of the (**d**) PTF, (**e**) DTF, and (**f**) TNP powders.

The morphological features of PTFs, DTFs, and TNPs were observed by FE-SEM and FE-TEM, as shown in Figure 1. Figure 1a shows PTF prepared using a spinning process, in which numerous pores in the structure were confirmed from the fiber surface and cross-sectioned inset image. The PS in the spinning solution was separated with PAN during electrospinning. Subsequently, PS in the as-spun fibers was decomposed into CO_2_ gas during heat treatment, which formed the numerous pores between TiO_2_ crystals in the structure. On the other hand, DTF fibers prepared from the solution without PS showed dense structure in Figure 1b. Without the effect of phase separation by adding PS, pores were not generated in the structure. Therefore, it was hard to observe any pores, even in the cross-sectioned inset image in Figure 1b. The mean fiber diameter of PTF and DTF measured from FE-TEM images were both 200 nm. From the elemental mapping images in Figure 1d,e, both TiO_2_ nanofibers showed uniformly distributed Ti and O elements over the nanostructure, but the C element was negligible, which proves the completely decomposition of C during heat treatment. The TNPs were rice-shaped and had an average particle size of about 30 nm (Figure 1c). Figure 1d–f shows the FETEM and EDX mapping results of the PTF, DTF, and TNP powders. The EDX mapping results show that only Ti and O were present in the samples. C was detected on the carbon tape used for the sampling. The EDX results also showed that the PTFs were less dense as compared to the DTFs. BET surface area measurements were carried out to determine the pore diameters and surface areas of the samples quantitatively.

**Figure 2:**
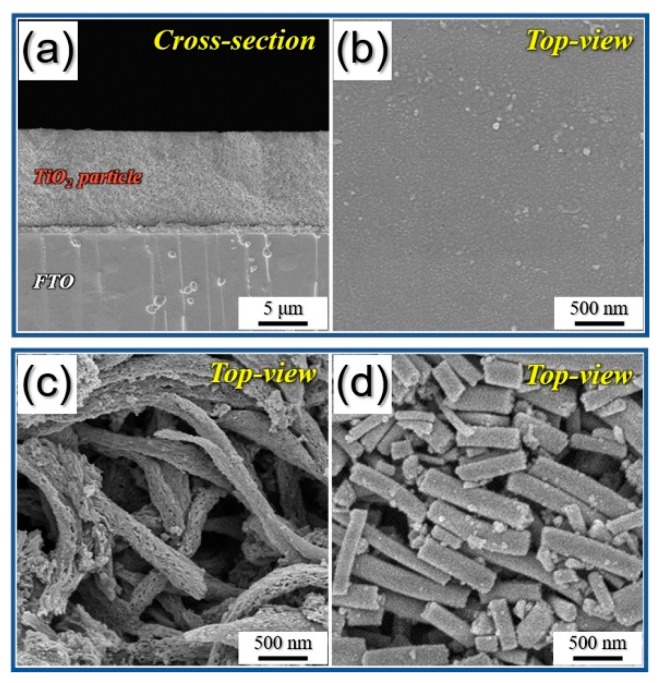
FESEM images of the (**a**) cross-section view of the TNP photoelectrode, and top-view of the (**b**) TNP, (**c**) TNP/PTF and (**d**) TNP/DTF photoelectrodes.

The pore diameter and surface areas of the PTFs, DTFs, and TNPs were measured by carrying out BET measurements. The pore distribution and diameter determine the fast charge transfer and light harvesting effect of porous fibers. However, a fiber structure is not good for the adsorption of light-absorbing dyes. Adsorption of dye molecules is closely related to the specific surface area of photoelectrode materials. Hence, in this study, we fabricated various photoelectrodes using the TNPs, PTFs, and DTFs. The TNP, PTF, DTF, TNP-PTF, TNP-DTF, TNP/PTF, and TNP/DTF photoelectrodes were used to assemble DSSCs. The photovoltaic properties of these DSSCs were investigated. Figure 2 shows the cross-section and top view of the TNP/PTF, TNP/DTF, and TNP photoelectrodes. The thickness of the TNP photoelectrode was about 8 ± 0.6 μm and the PTF and DTF light scattering layers were coated on it. The top-view of TNP/PTF and TNP/DTF (Figure 2c,d) revealed that TNP/PTF showed porosity and a loofah-like formation. These pores could improve the light-harvesting and electrolyte diffusion of the photoelectrode and hence improved the charge transport and accessibility of the electrolyte ions [28,29,30,31].

**Figure 3:**
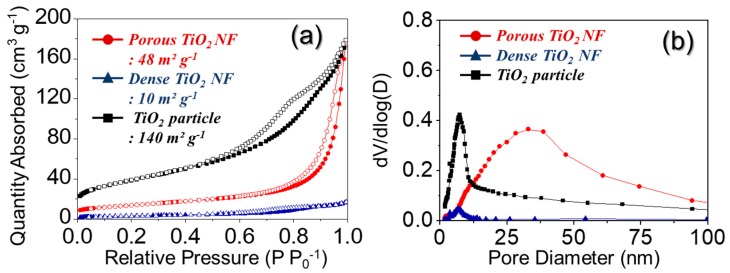
(**a**) N_2_ adsorption isotherms and (**b**) pore-size distribution of the PTF, DTF, and TNP powders.

N_2_ adsorption of all the samples was carried out and the results are shown in Figure 3 and Table 1. The TNPs exhibited a specific surface area of 140 m^2^ g^−1^. The surface areas of the PTFs and DTFs were lower than that of the TNPs. The TNPs, PTFs, and DTPs showed type-IV adsorption isotherms, indicating the presence of mesoporous structures with an average pore size of 7.13, 18.47, and 5.48 nm, respectively. In the case of the TNPs, the hysteresis loop was more prominent for the relative pressures (P/P_0_) in the range of 0.7–1.0, indicating the presence of many pores. Figure 3b shows the pore diameter distribution of the samples (within 0–100 nm). The PTFs showed a large mesopore volume of 10–50 nm. They exhibited a hierarchical pore structure consisting mainly of mesopores along with some micropores. Therefore, the use of PTF photoelectrodes can improve the electrode-electrolyte interactions and shorten the ionic transport paths, thus promoting the charge transport and enhancing the accessibility for electrolyte ions.

**Figure 4:**
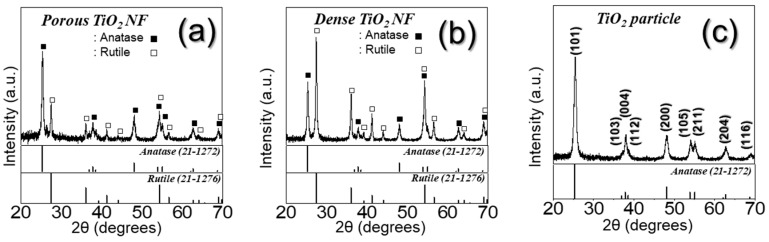
X-ray diffraction of the (**a**) PTF, (**b**) DTF, and (**c**) TNP powders.

The structural properties of the PTF, DTF, and TNP powders were investigated using their XRD patterns shown in Figure 4. The PTFs and DTFs showed mixed anatase (space group *I41*/*amd*, card no. 21-1272 in the JCPDS database) and rutile (card no. 21-1276 in the JCPDS database) phases. On the other hand, the TNPs showed only the anatase phase [32,33]. The dark and white squares in Figure 4 correspond to the anatase and rutile phases, respectively. The XRD results showed that the rutile phase fraction of the DTFs (rutile phase peak intensity was higher) was higher than that of the PTF. The rutile phase has a slightly lower bandgap than the anatase phase and can absorb a little sunlight in the near-UV region. The open-circuit voltage of DSSCs can be controlled by adjusting the position of the TiO_2_ conduction band. Most of the studies in this context have focused on anatase TiO_2_. However, rutile TiO_2_ is easy to produce and has superior light-scattering properties, which is beneficial for effective light-harvesting [34]. Therefore, we used the PTFs and DTFs (which consisted of the rutile phase) as light scattering layers for DSSCs.

**Figure 5:**
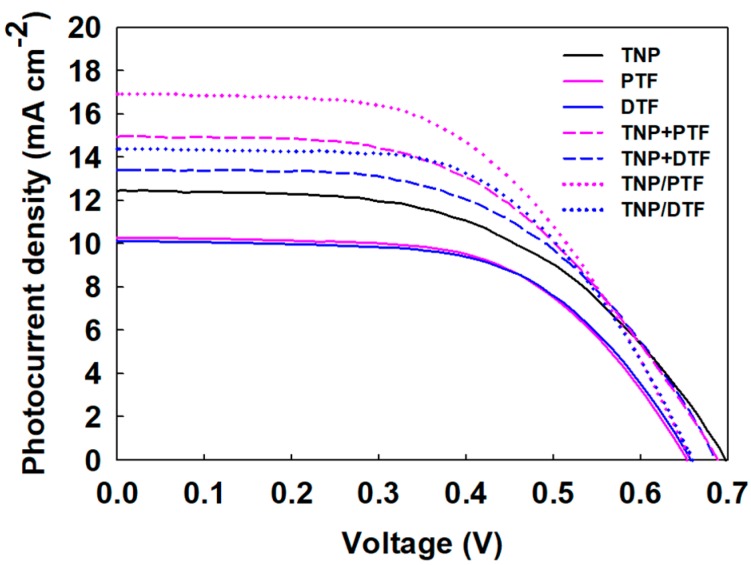
Photocurrent density–voltage curves of the DSSCs with different photoelectrodes.

Figure 5 shows the photocurrent density–voltage curves of the DSSCs fabricated using different photoelectrodes. As shown in Figure 5, the TNP photoelectrode showed a higher open-circuit voltage (*V_oc_*) (0.69 ± 0.02 V) than the PTF and DTF photoelectrodes. The *V_oc_* of DSSCs can be controlled by adjusting the position of the TiO_2_ conduction band. Hence, the *V_oc_* of the PTF and DTF photoelectrodes (having the rutile crystalline structure) was lower than that of the anatase TNP photoelectrode. In addition, the photocurrent densities of the PTF and DTF photoelectrodes were lower than that of the TNP photoelectrode because of the loading of a small amount of dye. Dye loading of a photoelectrode is closely related to the specific surface area of TiO_2_; the larger the specific surface area of TiO_2_, the larger is the amount of dye that can be adsorbed on the TiO_2_ surface. The addition of the PTFs and DTFs to the TNP photoelectrodes resulted in an increase in the short circuit photocurrent density (*J_sc_*) and PCE (*η*) because of the improved charge transport and enhanced accessibility for the electrolyte ions. On the other hands in order to improve the light harvesting effect of the DSSCs, the PTF or DTF light scattering layers were coated on the TNP photoelectrodes. As the result, the TNP/PTF photoelectrode showed the highest *J_sc_* and *η*. The high *J_sc_* can be attributed to the increased light harvesting effect due to the PTF light-scattering layer. The rutile phase-mixed PTF acted as an energy barrier and increased the physical separation between the injected electrons and oxidized dyes/redox couples, thereby retarding the recombination reactions in the resulting DSSCs [35]. The detailed photocurrent density–voltage results are given in Table 1. The TNP/PTF photoelectrode-based DSSC showed the highest *η* of 5.9 ± 0.13%, which is approximately 28% higher than that of the TNP photoelectrode-based DSSCs (4.6 ± 0.07%).

**Figure 6:**
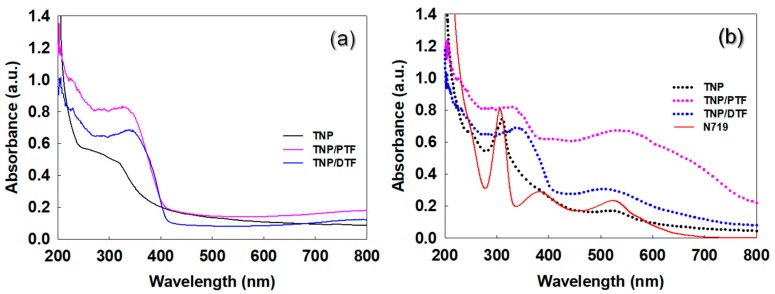
UV-Vis absorption spectra of the different photoelectrodes (**a**) before and (**b**) after N719 dye adsorption.

The UV-Vis absorption spectra of the TNP, TNP/DTF, TNP/PTF photoelectrodes, and N719 dye are shown in Figure 6. As shown in Figure 6a, all the samples showed strong absorption in the UV region (wavelengths lower than 250 nm) before dye adsorption. It can be observed that the absorption edges of the TNP/DTF and TNP/PTF photoelectrodes slightly red-shifted with respect to the TNP photoelectrode, suggesting that the TNP/DTF and TNP/PTF photoelectrodes caused a small decrease in the energy band gap. In addition, the TNP/DTF and TNP/PTF photoelectrodes showed an absorption peak in the near-UV region at 330 nm, which can be attributed to their rutile structure. All the samples showed almost no absorption in the visible region. As shown in Figure 6b, the N719 dye showed an absorption peak in the visible region at 530 nm. The intensity of this peak was high in the case of the TNP/PTF photoelectrode. This indicates that the use of the PTF light scattering layer (TNP/PTF) coating increased the visible region absorption of the DSSC.

## 4. Conclusions

Porous and dense TiO_2_ nanofibers (200 nm fiber diameter) were successfully prepared using electrospinning and were used as additives or light-scattering layers to enhance the photovoltaic properties of DSSC. Both the PTFs and DTFs showed mixed phases of anatase and rutile TiO_2_ crystals. The addition of 10 wt.% of PTFs or DTFs increased the short circuit photocurrent density (*J_sc_*) of the TNP photoelectrode because of an improvement in the charge transport and accessibility for the electrolyte ions. In addition, the TNP/PTF photoelectrode improved the visible light absorption of the DSSC because of the mountainous nature of light due to the PTF light scattering layer. The highest short circuit photocurrent density was shown by the TNP/PTF photoelectrode (16.96 ± 0.79 mA cm^−2^). The TNP/PTF photoelectrode showed a high PCE of 5.9 ± 0.13% and an open-circuit voltage (*V_oc_*) of 0.66 ± 0.02 V. These results show that the use of scattering layers made up of PTFs is an effective approach to improve the photovoltaic performance of DSSCs.

## Figures and Tables

**Table 1:** BET and crystal structure of the TNP, PTF, and DTF powders and the photovoltaic parameters of different photoelectrode used DSSC.

Photo-Electrodes	BET (m^2^ g^−1^)	Pore Size (nm)	Pore Volume (cm^3^ g^−1^)	Crystal Structure	*V_oc_* (V)	*J_sc_* (mA cm^−2^)	*FF* (%)	*η* (%)
TNP	140.08	7.13	0.2763	Anatase	0.69 ± 0.02	12.51 ± 0.60	53 ± 2	4.6 ± 0.07
PTF	48.01	18.47	0.2794	Anatase, Rutile	0.65 ± 0.01	10.30 ± 0.96	56 ± 2	3.8 ± 0.19
DTF	23.16	5.48	0.0460	Anatase, Rutile	0.66 ± 0.01	10.18 ± 1.24	48 ± 7	3.2 ± 0.15
TNP-PTF	-	-	-	-	0.68 ± 0.01	14.95 ± 1.03	54 ± 2	5.4 ± 0.17
TNP-DTF	-	-	-	-	0.68 ± 0.01	13.42 ± 0.50	55 ± 1	5.1 ± 0.16
TNP/PTF	-	-	-	-	0.66 ± 0.02	16.96 ± 0.79	56 ± 2	5.9 ± 0.13
TNP/DTF	-	-	-	-	0.66 ± 0.01	14.39 ± 0.50	54 ± 1	5.2 ± 0.13
